# Cancer derived exosomes induce macrophages immunosuppressive polarization to promote bladder cancer progression

**DOI:** 10.1186/s12964-021-00768-1

**Published:** 2021-09-14

**Authors:** Ziming Jiang, Yiming Zhang, Yu Zhang, Zhankui Jia, Zhengguo Zhang, Jinjian Yang

**Affiliations:** Department of Urology, The First Affiliated Hospital of Zhengzhou University, Zhengzhou, 450052 Henan China

**Keywords:** Exosomes, Bladder cancer, Tumor associated macrophages, Tumor microenvironment

## Abstract

**Background:**

Exosomes mediated crosstalk between tumor cells and other stromal cells including tumor associated macrophages plays an essential role in reprogramming tumor microenvironment (TME) to facilitate tumor progression. However, the mechanism of tumor derived exosomes promotes bladder cancer progression have not been defined.

**Methods:**

Exosomes were extracted from bladder cancer cells MB49 conditioned medium by ultracentrifugation. The effects of MB49-derived exosomes on macrophages polarization were analyzed by qPCR, flow cytometry, and Western blot. The immunosuppressive phenotype and function of MB49-derived exosomes stimulated macrophages were verified by tumor xenograft assays and T cell co-culture experiments. Exosomal miRNAs were analyzed by microarray to identify potential targets regulating macrophage polarization.

**Results:**

MB49-derived exosomes could be ingested by macrophages, consequently promoting macrophages immunosuppressive polarization. Mechanically, the MB49-derived exosomes induced macrophage M2 polarization was mediated by down-regulation of PTEN and activation of AKT/STAT3/6 signaling. Moreover, hindrance of the generation or secretion of exosomes by GW4869 inhibited macrophages differentiation into immunosuppressive phenotype and function, thereby suppressed tumor growth in a mouse subcutaneous tumor model.

**Conclusion:**

Our study confirmed the contribution of bladder cancer derived exosomes on the establishment of immunosuppressive TME and provided a potential therapeutic target for bladder cancer treatment.

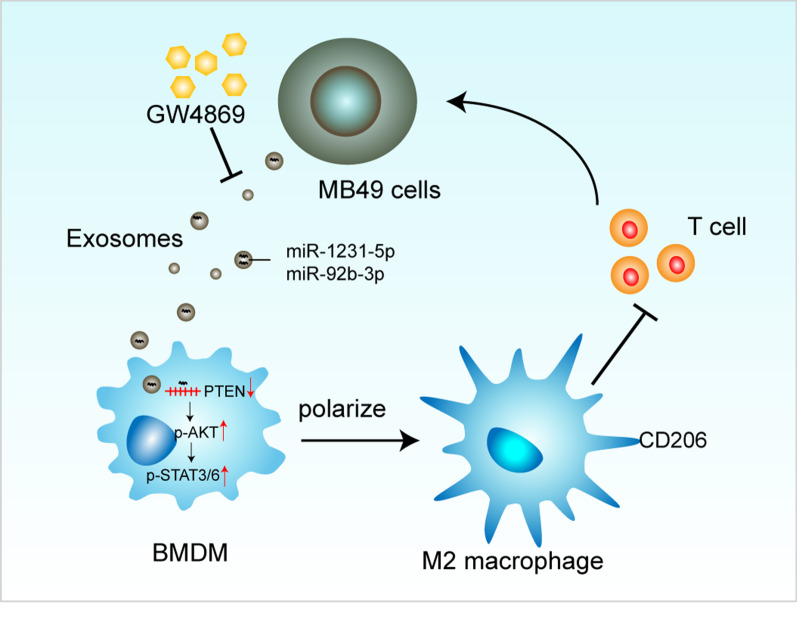

**Video Abstract**

**Supplementary Information:**

The online version contains supplementary material available at 10.1186/s12964-021-00768-1.

## Background

Bladder cancer is one of the most common type of cancers, with more than 357,000 new cases and 130,000 deaths worldwide each year [1]. About 75% bladder cancer is non-muscle-invasive at diagnosis, and the remainder present as muscle invasion with/out metastasis. Even following effective treatment like transurethral resection of bladder cancer and intravesical chemotherapy, part of non-muscle-invasive bladder cancer can progress to muscle invasion [2]. The muscle invasive bladder cancer (MIBC) without metastasis is managed by radical cystectomy with/out neoadjuvant chemotherapy, however it has high recurrence and metastasis rate. The five-year survival rate of MIBC is less than 70%, even worse for metastatic form [3, 4]. Therefore, it is of particular importance to clarify the mechanisms of bladder cancer progression and identify new targets for bladder cancer therapy.

The tumor microenvironment (TME) refers to the environment around a tumor, consisting of tumor cells, immune cells, fibroblasts, the surrounding blood vessels and the extracellular matrix, which plays an essential role in cancer development, progression and control [5]. Since the immune cells are important determinants of TME, the interplay between host immune system and tumor cells has been intensively investigated in the past decade. Recently, immunotherapy has been used for a variety of cancers, but still far from meeting clinical needs [6]. Tumor associated macrophages (TAM), a major component of immune cells in TME, have been suggested to play a central role in tumor progression [7–9]. TAM is generally considered to be immunosuppressive with high *CD206/CD163* expression [10, 11]. Increasing evidence indicates that TAM could increase tumor invasion and metastasis by taming host adaptive immunity [12, 13]. It is believed that re-educating TAM to tumor killing function may potentially contribute to bladder cancer therapy. Therefore, it is urgent need to understand how TAM polarization in bladder TME during tumor progression.

Emerging evidence has suggested that the communication between tumor cells and macrophages plays a key role in mediating TAM polarization [14, 15]. Signals originated from tumor cells such as transforming growth factor beta (TGF-β), could promote differentiation of non-activated macrophages into a TAM-like immunosuppressive phenotype characterized as increased anti-inflammatory cytokine and decreased pro-inflammatory cytokine expression [11, 16]. Exosomes, small vesicles derived from cells, are wrapped in a lipid bilayer that carry various biological molecules, including proteins, polysaccharides, lipids, DNA, RNA, metabolites, and mediate the information transmission and exchange of material between cells [17, 18]. Therefore, exosomes contribute to information and material exchange between tumor cells and other stromal or immune cells in TME [19]. It has been reported that exosomes originated from TAM could promote metastasis of tumor cells [20] as well as resistance to chemotherapy [21]. Reciprocally, exosomal miR-21 secreted by bladder cancer cell T24 cells could polarize THP-1 cell-derived macrophages into the M2 phenotype by activating PI3K-AKT-STAT3 signaling pathway [22]. However, the underlying mechanism of tumor cells derived exosomes mediating macrophage polarization is still not fully understood.

In current study, we aimed to investigate how bladder tumor cells derived exosomes induce macrophage polarization and the effects on tumor growth. We first investigate the effect of mouse bladder cell MB49-derived exosomes on bone marrow-derived macrophages (BMDM) polarization, as well as the influence of MB49-derived exosomes stimulated macrophages on T cell proliferation. Moreover, we also studied the potential therapeutic function of GW4869 on bladder cancer growth by inhibiting the generation or release of exosomes.

## Materials and methods

### Cell culture

The mouse bladder cancer cell line MB49 was obtained from the Chinese Academy of Sciences. MB49 cells were cultured in DMEM medium (GE Healthcare Life Sciences, Pittsburgh, PA, USA), supplemented with 10% fetal bovine serum (FBS; Gibco; Thermo Fisher Science, Massachusetts, USA).

### Animals

Female C57BL/6J mice were 8–10 weeks of age and purchased from the Vital River Laboratory Animal Technology Company (Beijing, China). All the mice were housed in IVC cages on racks in a room with controlled temperature (20–25 °C) and humidity (40–60%), and were subjected to 12 h light/dark cycles in a specific pathogen-free (SPF) facility. The animal experiment was approved by the Ethical Review Committee of the First Affiliated Hospital of Zhengzhou University.

### BMDM isolation

Bone marrow-derived macrophages (BMDM) were isolated from the femur and tibia of adult male mice according to our previous publication [23]. Briefly, mouse femoral tissue was isolated, and the bone marrow was flushed with 3 ml of normal saline (NS). After red blood cell lysis, bone marrow cells were suspended at 2–4 × 10^6^ cells/ml in RPMI-1640 medium, supplemented with 10% FBS and 30 ng/ml GM-CSF (PEPROTECH Inc., Rocky Hill, USA). BMDM were cultured in a humidified incubator at 37 °C and 5% CO2.

### Isolation and identification of exosomes

MB49 cells were washed twice with PBS when its confluence reach to 90%, following cultured with serum-free RPMI 1640 medium for 24 h. The culture supernatant was collected and centrifuged at 300*g* and 15,000*g* for 20 min respectively, which is to remove the suspended cells and cell debris. Then the collected supernatant was subjected to ultracentrifugation at 100,000*g* for 70 min at 4 °C (HITACHI ultracentrifuge, CS150FNX). The obtained exosome pellet was washed with PBS and concentrated after ultracentrifugation, which is to improve the purification of exosomes. The MB49-derived exosomes were verified by transmission electron microscopy (HT7800, HITACHI) and the expression of exosome specific markers CD63 and HSP90 by Western blot analysis.

### RNA extraction and qRT-PCR

The total RNA of exosomes stimulated BMDM was extracted with Trizol® reagent (Invitrogen, Carlsbad, CA, USA), and reverse transcription was performed with PrimeScript™ RT Master Mix (Takara Co. Ltd.). Real-time PCR was performed on the ABI PRISM 7300HT sequence detection system (Applied Biosystems, Foster, CA, USA) using SYBR Green PCR Master Mix (Application Takara, Otsu). The relative expression level of mRNA was calculated by the 2^−ΔΔCt^ method. The sequences of all primers are shown in the Additional file 1: Table S1. Each experiment was repeated three times.

### Western blot

BMDM were lysed with RIPA buffer (Beyotime institute of Biotechnology, Shanghai, China). The bicinchoninic acid protein assay (BCA) kit (Beijing Leagene Biotech co. Ltd) was used to determine the protein concentration. Total 20 μg protein were separated on SDS-PAGE gel and transferred to 0.45 μm PVDF membrane. The membrane was blocked with 5% skimmed milk at room temperature for 1 h. The primary antibodies were incubated overnight at 4 °C. The peroxidase-conjugated secondary antibody was incubated for 60 min at room temperature. The protein bands were then visualized using an enhanced chemiluminescence kit (Beyotime institute of Biotechnology) and scanned by an imaging system (Bio-Rad Laboratories, Inc. Hercules, CA, USA). The ImageJ software v1.8.0 (National Institutes of Health) was used to quantify the density measurement. The primary and secondary antibodies used in this study were listed in Additional file 2: Table S2.

### Exosomes tracing

To detect the uptake of MB49-derived exosomes by BMDM, the fluorescent dye azide cyanine-Cy5.5 (0.2 μM, Fanbo BioChemical, Beijing, China) was added to the exosomes at 37 °C for 30 min, following the Cy5.5 labeled exosomes were centrifuged at 100,000*g*, 4 °C for 70 min to remove residual dye, and then incubated with BMDM for 3 h in the dark at 37 °C. The nuclei were stained with DAPI (Beyotime institute of Biotechnology, Shanghai, China) at room temperature for 5 min, and then observed with a laser scanning confocal microscope or analyzed by flow cytometry analysis. For tracking the uptake of circulating MB49-derived exosomes in vivo, we first injected Cy5.5 labeled exosomes (1 μg in 100 μl PBS) into the mouse via the tail vein. After 12 h, the bladder was harvested and digested with 0.1 mg/ml collagenase D / DNase I (100 U/ml) (Sigma-Aldrich, Inc. St. Louis, MO, USA) solution. The Cy5.5 positive cells in CD45^+^F4/80^+^CD11b^+^ macrophages were analyzed by flow cytometry.

### Splenic T cell isolation

T cells were isolated from the spleen of 8 weeks female C57BL/6J mice. Briefly, single cell suspension of spleen was obtained by injecting 5 ml PBS, subsequent dissection and passing through a 70 μm filter (BD Bioscience, 352350, Florida, USA). The RBC lysis buffer (R1010, Solarbio life science, Beijing, China) was used to remove red blood cells. The splenic cells were suspended in complete RPMI-1640 medium and cultured for 60 min. The unattached T cells were collected and washed with PBS, and then stained by adding 2.5 μM Carboxyfluorescein succinimidyl ester (CFSE, abcam, 113853) for 10 min for following co-culture experiments.

### T cell co-culture experiments

MB49 cells were treated with/without 10 μM GW4869 (MCE, HY-19363) for 24 h. Afterwards, the exosomes were collected from culture supernatant by ultracentrifugation. After stimulating BMDM with exosomes for 48 h, CFSE-labeled splenic T cells were added and co-cultured for another 3 d. Then the percentage of CD4^+^/CD8^+^ T cells was detected by flow cytometry.

### Flow cytometry

To detect the effects of MB49 conditional medium (CM) and MB49-derived exosomes on BMDM, the freshly isolated BMDM were stimulated with MB49 CM or MB49-derived exosomes for 48 h. Then BMDM were collected and stained with CD45, F4/80, CD206 and CD11b. For the tumor associated immune cells identification, the MB49 derived subcutaneous tumor tissues were taken and digested with 0.1 mg/ml type D collagenase (Sigma-Aldrich, Inc. St. Louis, MO, USA) for 30 min at 37 °C, and the single cell suspension were filtered and stained with macrophage markers (CD45, F4/80, CD206 and CD11b). The macrophage population was detected by using flow cytometer (BD Canto, Franklin Lakes, USA), and data were analyzed by FlowJo X (Tree Star, Ashland, OR, USA) software. Each experiment was repeated three times. The used antibodies were listed in Additional file 3: Table S3.

### Microarray analysis of exosomal miRNAs

To determine the miRNAs contained in MB49-derived exosomes, a microarray analysis using Agilent mouse miRNA microarray kit (Agilent Technologies) was performed (OE Biotech Company, Shanghai, China). The sample preparation, miRNAs labeling, microarray hybridization and washing processes were carried out according to the manufacturer’s protocols. Data extraction and visualization were performed using Feature extraction software (version 10.7.1.1, Agilent Technologies).

### Tumor xenografts

To evaluate the influence of exosomes to macrophage polarization in vivo, we established a mouse subcutaneous tumor model. Briefly, total 1 × 10^6^ MB49 cells were transplanted subcutaneously into the right flank of a male 8–10 week-old mouse. From day 3, GW4869 (2.5 μg/g in DMSO/saline) was intraperitoneally injected in every 3 days for total 4 times. A sham control group was injected with DMSO/saline alone. Tumor volumes were calculated as 1/2 × length × width^2^. The mice were sacrificed on day 15 and the tumor/body weight ratio was measured. The animal experiment was approved by the Ethical Review Committee of the First Affiliated Hospital of Zhengzhou University.

### RNA interference

The mimics and inhibitors of miR-1231-5p and miR-92b-3p as well as their negative controls were purchased from Shanghai Biotechnology Corporation (Shanghai, China). The sequences of miRNA inhibitor and mimics were listed in Additional file 3: Table S3 and Additional file 4: Table S4. Transfection of miRNAs into BMDM were conducted using the LipoHigh liposome efficient transfection reagent (Shanghai Biotechnology Corporation), according to the manufacturer’s instructions.

### Statistical analysis

The statistical analysis was performed using GraphPad Prism 8 software. Comparisons between two groups were made using *t-test* as appropriate. For multiple comparisons, one-way ANOVA was applied. Statistically significant differences are indicated as follows: *P < 0.05; **P < 0.01; ***P < 0.001.

## Results

### MB49 cells conditioned medium induces macrophages differentiation into M2 phenotype

**Fig. 1:**
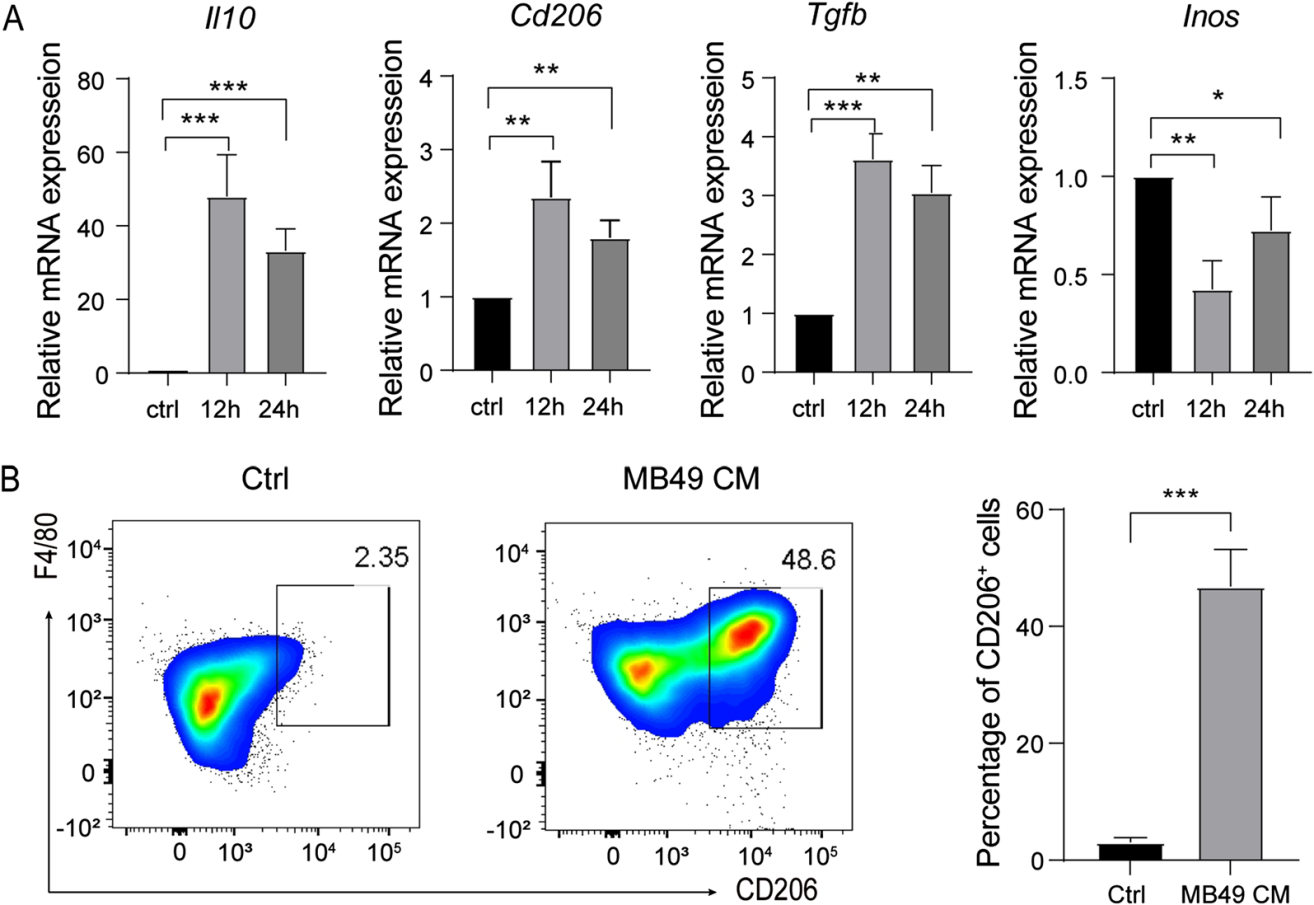
MB49 cells conditioned medium could promote the transformation of BMDM to M2 phenotype. **A** The expression of *Il-10, Cd206, Tgfb* and *Inos* was quantified by qRT-PCR in macrophages stimulated with MB49 cells conditioned medium for 12 and 24 h, respectively. Results are present as the means ± SD of 3 independent experiments. Data were analyzed by one-way ANOVA with multiple comparisons test (*P < 0.05, **P < 0.01, ****P < 0.0001). **B** BMDM were stimulated with MB49 conditioned medium for 48 h, and the proportion of F4/80^+^CD206^+^ cells was detected by flow cytometry. Representative images of each sample are shown. Data were analyzed by t-test (Mean ± SD, n = 3, ***P < 0.001)

Previous studies have suggested that secreted factors from tumor cells could mediate the polarization of macrophages [24, 25]. In order to investigate the effect of bladder cancer cells secreted factors on macrophage polarization, mouse BMDM were treated with bladder cancer cell MB49 cells culture supernatant and gene expression was analyzed by RT-qPCR. The expressions of M2 related genes, including interleukin-10 (*Il-10*), *Cd206* and transforming growth factor beta (*Tgfb*) were increased, whilst M1 gene *Inos* (Fig. 1A) was decreased. The MB49 cells culture supernatant mediated macrophages M2 polarization was further verified by showing the increased F4/80^+^ CD206^+^ macrophage population (Fig. 1B). These results indicated that MB49 cells conditioned medium (CM) can promote the polarization of BMDM to M2 phenotype.

### Identification of exosomes derived from MB49 cells and ingested of exosomes by macrophages in vitro and in vivo

**Fig. 2:**
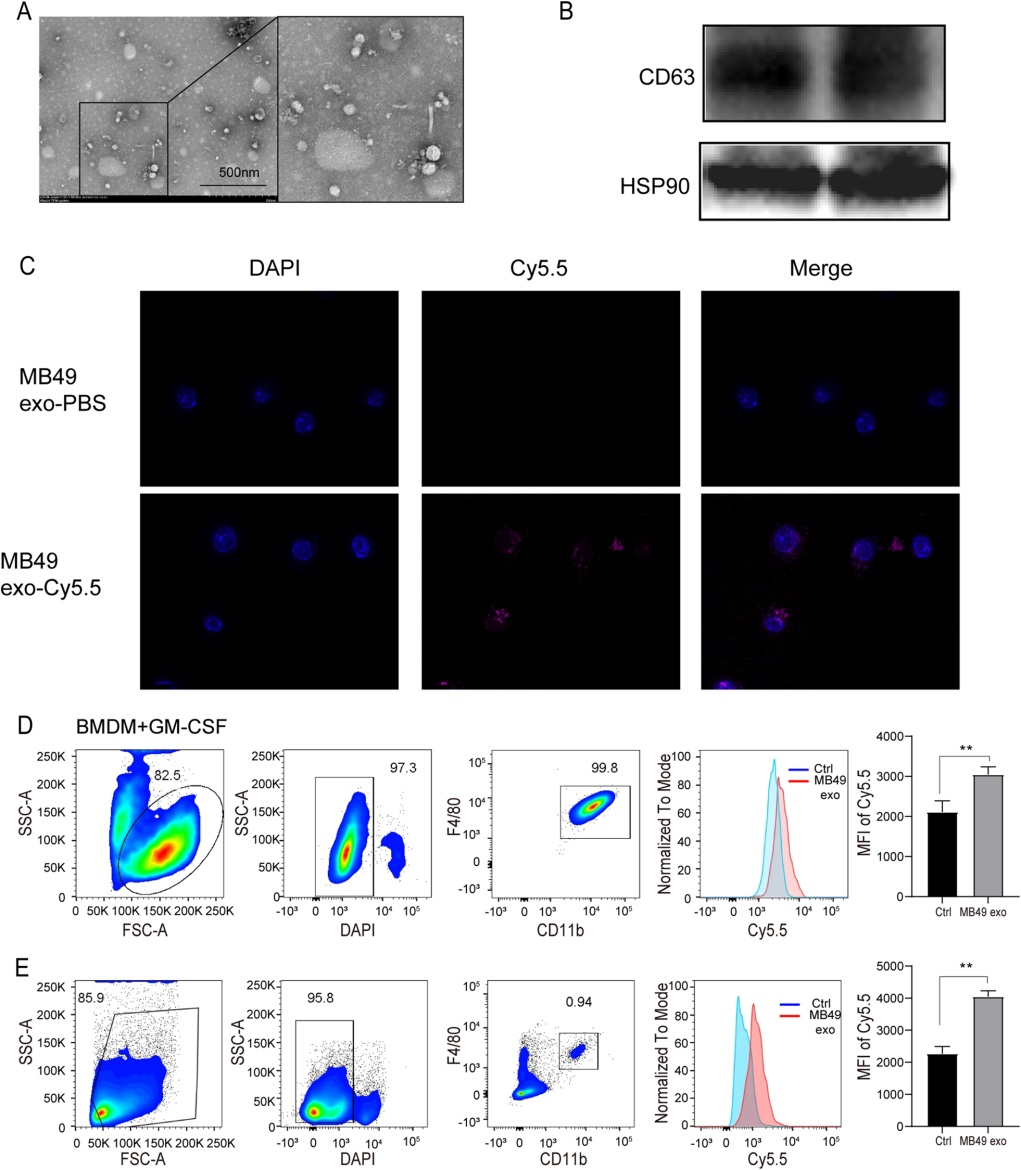
Identification of MB49-derived exosomes and their uptake in macrophages. **A** The morphology of exosomes isolated from MB49 cells were observed by electron microscope. Shown in the box are typical exosome particles. Scale bar, 500 nm. **B** The expression of CD63 and HSP90 were detected by Western blot analysis in MB49 exosomes. **C** Cy5.5-labeled exosomes were co-cultured with BMDM for 3 h, and the uptake of exosomes was observed under a confocal microscope. The representative images were shown. Scale bar, 10 μm. **D** The mean fluorescence intensity (MFI) of Cy5.5-labeled exosomes treated BMDM was measured by flow cytometry, and the control group was treated with PBS-exosomes. **E** Cy5.5-labeled MB49-derived exosomes or PBS MB49-exosomes were injected into mice via the tail vein. And the MFI of Cy5.5 labeled exosomes in CD45^+^F4/80^+^CD11b^+^ bladder macrophages was analyzed by flow cytometry. Each experiment was repeated three times, and the data were expressed as mean ± SD, t-test, **P < 0.01

We further asked if exosomes in MB49 cells CM could mediate macrophages M2 polarization. Exosomes were extracted by ultracentrifugation and the morphology was observed through an electron microscope (Fig. 2A). In addition, the expression of exosome markers HSP90 and CD63 was also detected by Western blot (Fig. 2A). To interrogate whether macrophage can uptake MB49-derived exosomes, BMDM were co-cultured with the Cy5.5 labeled exosomes. Immunofluorescence and flow cytometry analysis demonstrated that MB49-derived exosomes can be ingested by macrophages (Fig. 2C, D). In order to further study the possibility of exosomes enrichment and absorption in bladder macrophages, we injected Cy5.5-labeled MB49-derived exosomes into mice via the tail vein. The results of flow cytometry showed that the average fluorescence intensity of Cy5.5 in bladder F4/80^+^ CD11b^+^ macrophages was much higher than that of the control group (Fig. 2E). Based on the above results, we confirmed that exosomes originated form MB49 cells can be ingested by macrophages both in vitro and in vivo.

### MB49-derived exosomes mediated the polarization of macrophages into immunosuppressive phenotype

**Fig. 3:**
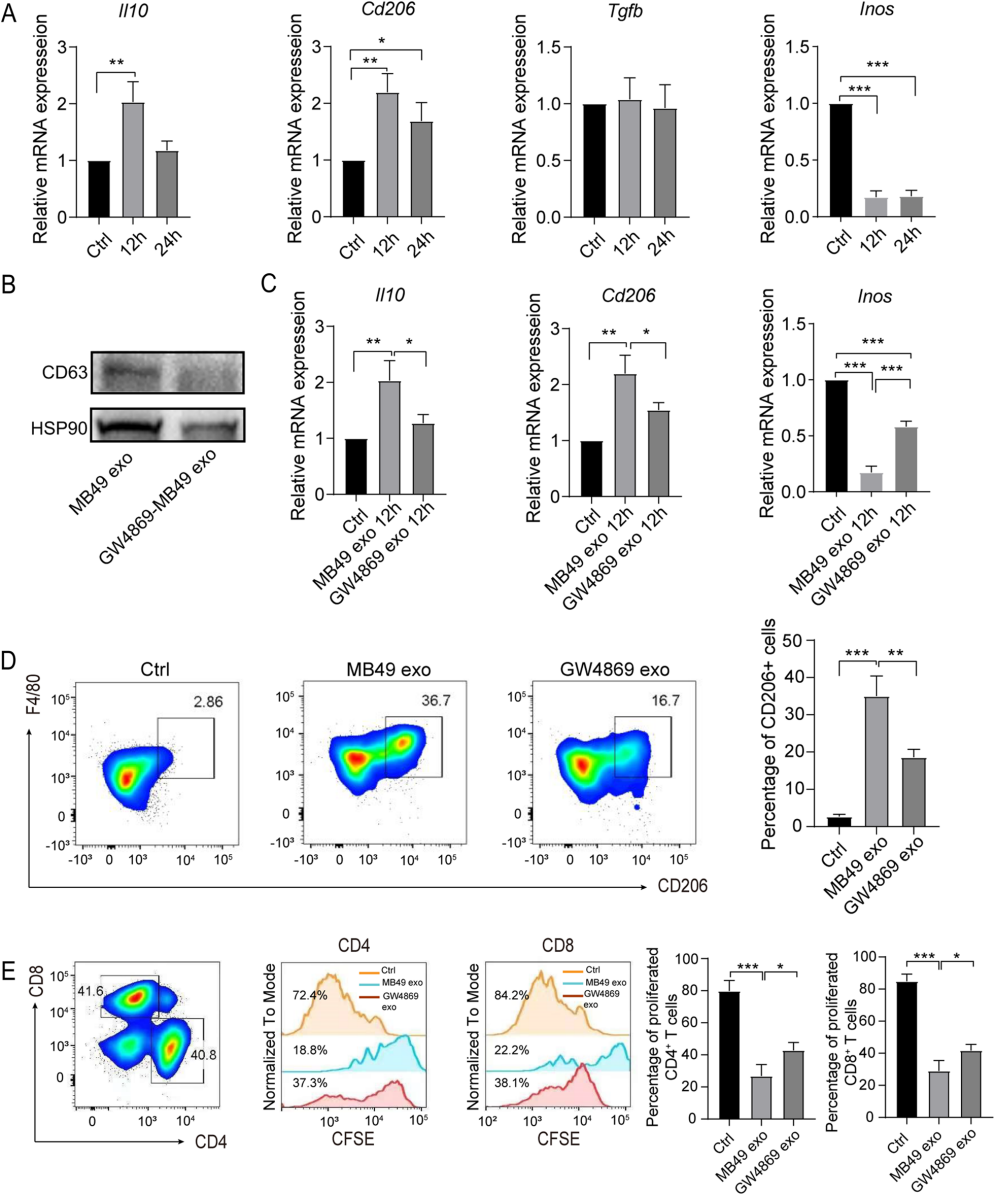
MB49-derived exosomes play a major role in inducing the immunosuppressive function of macrophages. BMDM were treated with MB49-derived exosomes, which were extracted from MB49 CM ± GW4869, for 12 h or 24 h respectively. **A** The expression of *Il10*, *Cd206*, *Tgfb* and *Inos* qRT-PCR was measured by qRT-PCR. **B** The expression of CD63 and Hsp90 was analyzed by Western blot. **C** The expression of *Il10*, *Cd206* and *Inos* was quantified by qRT-PCR. **D** BMDM were stimulated with exosomes for 48 h and flow cytometry were used to detect the percentage of F4/80^+^CD206^+^ macrophages, the representative plot was shown. **E** The exosome stimulated BMDM were further co-cultured with CFSE labeled splenic T cells for 3 d, the percentage of CFSE^+^ CD4^+^ or CD8^+^ T cells was measured by flow cytometry. The experiments were repeated for 3 times. Data are present as mean ± SD, one-way ANOVA, *P < 0.05; **P < 0.01; ***P < 0.001

To further identify the effect of MB49-derived exosomes on BMDM phenotype and function, we first detected the gene expression level of M1/M2 macrophage markers in MB49-derived exosomes treated BMDM. RT-qPCR results showed that the expression of *Il10*, *Cd206* and *Tgfb* in MB49-derived exosomes stimulated BMDM was significantly increased, whilst the expression of M1 macrophage gene *Inos* was reduced, indicating MB49-derived exosomes could polarize macrophage to M2 phenotype (Fig. 3A). To further confirm the polarization of M2 macrophages was exosomes dependent, we utilize GW4869, an exosome inhibitor, to detect the expression of M1/M2 macrophage markers. As expected, GW4869 treatment significantly reduced exosomes release, as showing reduced CD63 and HSP90 expression in MB49-derived exosomes by Western blot (Fig. 3B). Moreover, GW4869 treatment could inhibit MB49-derived exosomes mediated M2 macrophage gene *Il10* and *Cd206* upregualtion and increase M1 macrophages gene *Inos* expression (Fig. 3C). Additionally, the proportion of F4/80^+^CD206^+^ macrophages was significantly decreased in GW4869 treatment group comparing to MB49-derived exosomes group (Fig. 3D). To further investigate the immunosuppressive function of MB49-derived exosomes polarized macrophages, we co-cultured the MB49-derived exosomes stimulated BMDM with CFSE-labeled splenic T cells for 3 days. MB49-derived exosomes polarized BMDM significantly reduced the proliferation of CD4^+^/CD8^+^ T cells, whilst GW4869 treatment reversed the reduction of T cell proliferation (Fig. 3E). Collectively, these results indicate exosomes in MB49 CM play a dominant role in polarizing macrophages into M2 phenotype and immunosuppressive function.

### MB49-derived exosomes promote bladder tumor cells growth in a mouse subcutaneous tumor model.

**Fig. 4:**
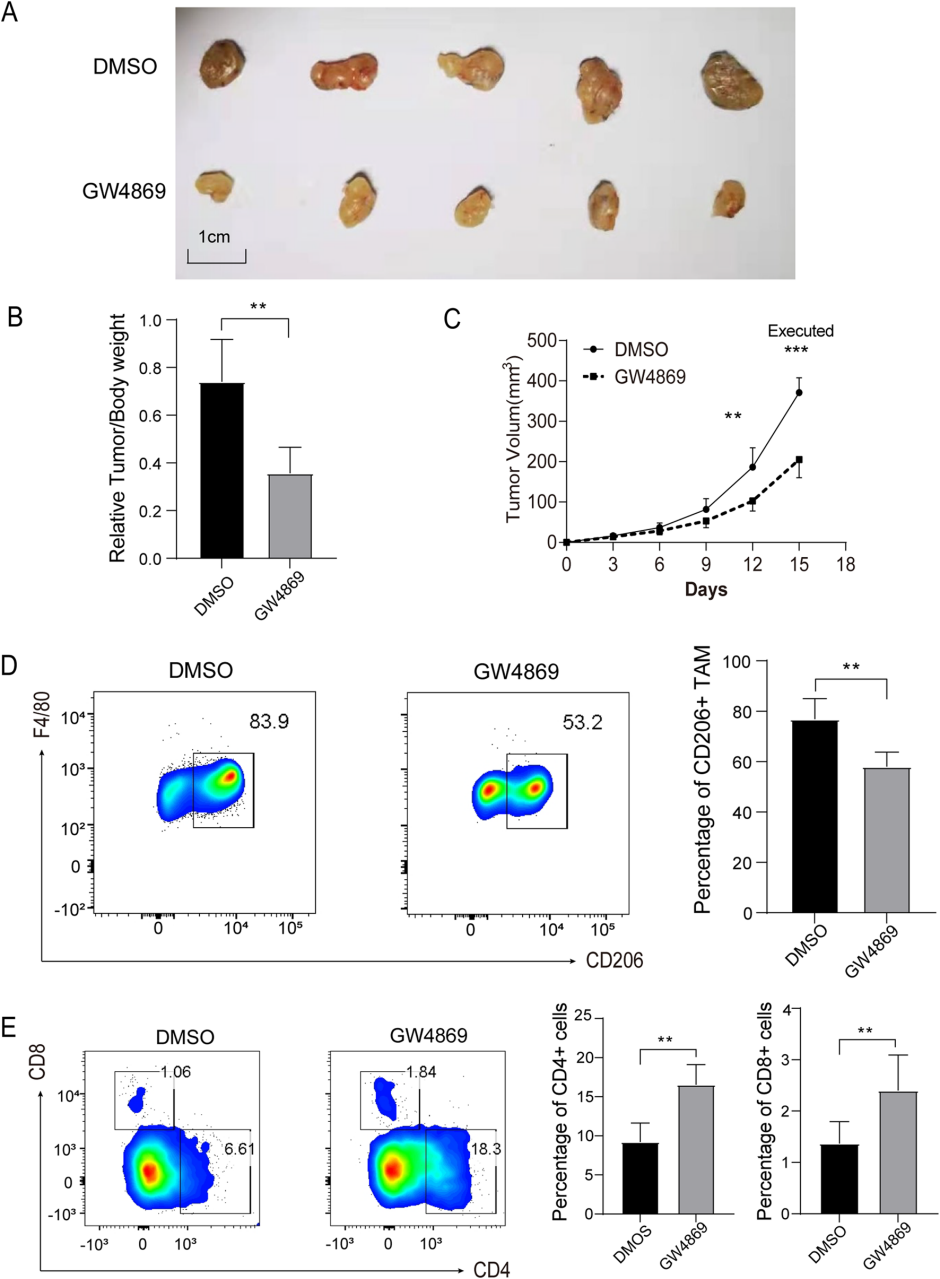
MB49-derived exosomes promote the growth of tumor cells. **A**–**C** Total 1 × 10^6^ MB49 cells were subcutaneously injected to mice, DMSO or GW4869 (2.5 μg/g) were intraperitoneally injected every three days (n = 10, 5 mice in each group). **A**, **B** Mice were sacrificed at 15 d, and the ration of tumor/body weight were measured. **C** The tumor volume was measured every three days and the growth curve was shown. **D**,** E** The proportion of CD45^+^F4/80^+^CD206^+^ macrophage and CD4^+^/CD8^+^ T cells were measured by flow cytometry in tumors (n = 5). T-test; Mean ± SD, *P < 0.05; **P < 0.01; ***P < 0.001

We next ask if the MB49-derived exosomes induced immunosuppressive macrophages could influence bladder tumor cells growth in vivo. In an established mouse subcutaneous tumor model, the tumor / body weight ratio and growth rate of xenograft tumors were significantly smaller and slower in GW4869 treatment group comparing to DMSO treatment group (Fig. 4A–C). Moreover, the majority of macrophages in tumors are M2 (F4/80^+^ CD206^+^) phenotype, and GW4869 treatment significantly reduced the percentage of M2 macrophages (Fig. 4D). Furthermore, the proportion of CD4^+^ and CD8^+^ T cells were significantly higher in GW4869 treatment (Fig. 4E). Taken together, these results indicate that MB49-derived exosomes can promote the growth of tumor cells by exosomes mediated immunosuppressive TME.

### Exosomal miRNAs promote the polarization of macrophages in a PTEN/p-AKT/STAT3/6 dependent manner

**Fig. 5:**
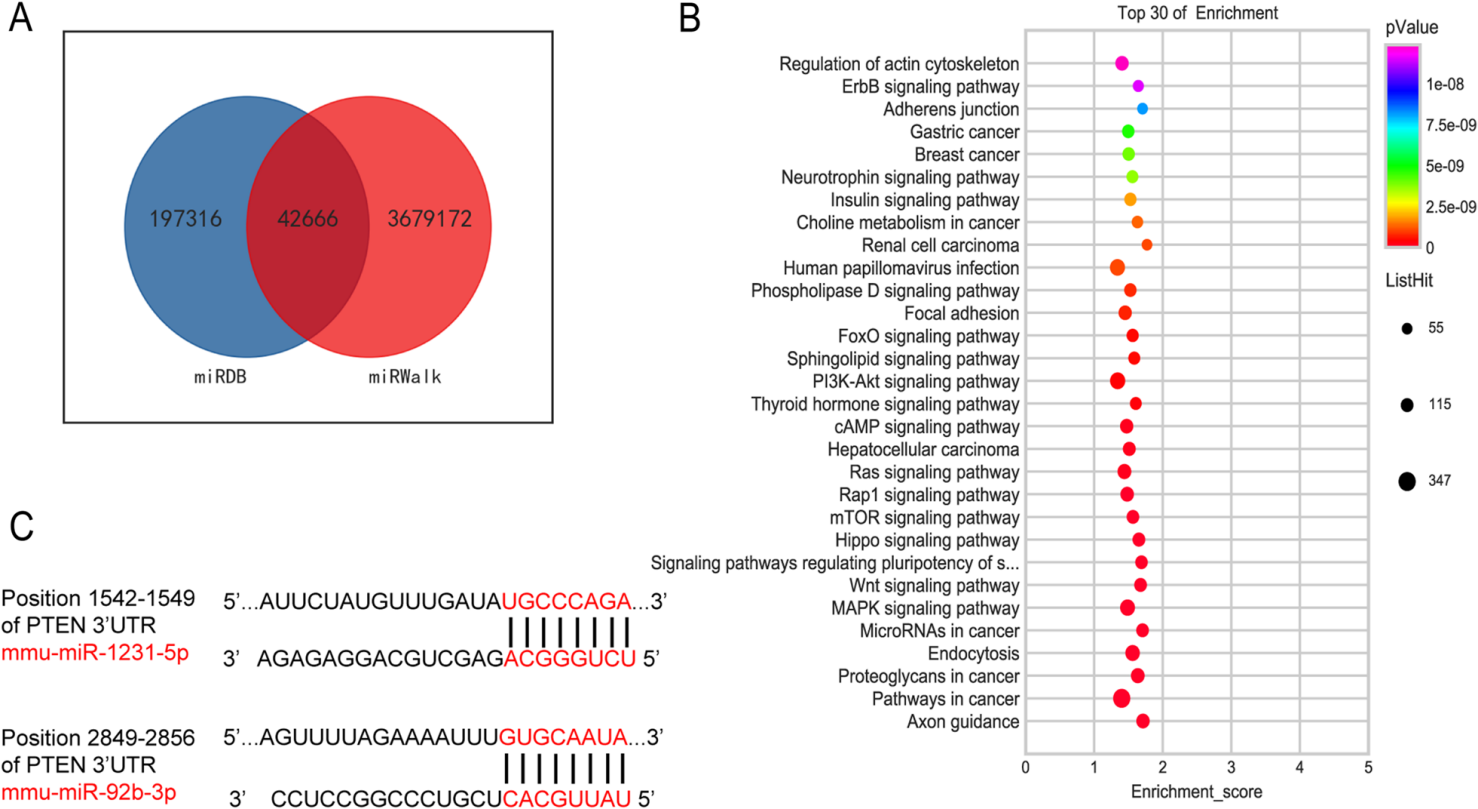
Microarray analysis and identification of PTEN target miRNAs in MB49-derived exosomes. **A** 42666 miRNAs were identified by microarray analysis in MB49-derived exosomes. **B** Bubble plot of GO enrichment analysis of target genes of MB4- exosmomal miRNAs. **C** The binding sites between miRNAs and *Pten* were shown

A growing body of evidence suggests that exosomal miRNA has multiple functions such as facilitating tumor growth, invasion, modulating TME [26]. To further elucidate miRNA profile in MB49-derived exosomes, microarray analysis was conducted. By analyzing two databases, 246 miRNAs were detected and 42666 target genes were predicted (Fig. 5A). Based on MB49-derived exosomal miRNAs target genes, a variety of cancer related signaling pathways like PI3K-AKT pathway were identified by KEEG analysis (Fig. 5B). Since PTEN is a tumor suppressor and negatively regulates PI3K-AKT signaling [27], two miRNAs, miR-92b-3p and miR-1231-5p were identified which can target *Pten* mRNA sites by using the online miRNA-mRNA matching tool Target Scan (Fig. 5C).

**Fig. 6:**
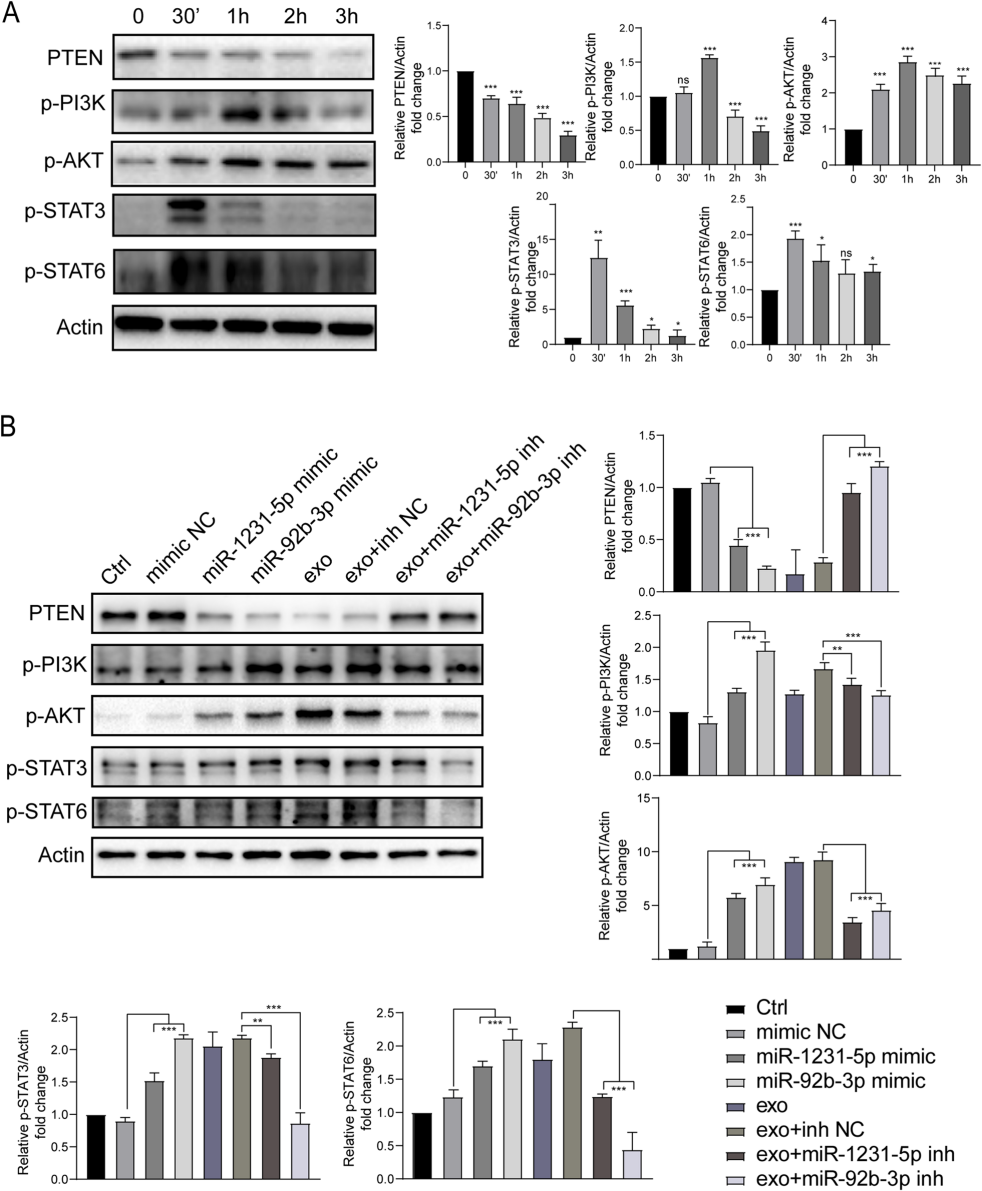
MB49-derived exosomes activate PTEN/AKT/STAT3/6 signaling pathways in BMDM. **A** BMDM were treated with MB49-derived exosomes for indicated time points, and the expression of PTEN, p-PI3K, p-AKT, p-STAT3 and p-STAT6 was analyzed by Western blot. **B** BMDM were stimulated with miR-92b-3p mimic or miR-1231-5p mimic or each inhibitor with MB49-derived exosomes for 3 h, respectively. The expression of PTEN, p-PI3K, p-AKT, p-STAT3 and p-STAT6 were detected by Western blot. Actin were used as a loading control. The experiments were performed three times and the relative protein ratio were analyzed by image J software. One way ANOVA, Mean ± SD, *P < 0.05; **P < 0.01; ***P < 0.001

In addition, Western blot results corroborate that the expression of PTEN was reduced in MB49-derived exosomes stimulated BMDM (Fig. 6A). Consequently, p-PI3K and p-AKT were significantly upregulated (Fig. 6A). Of note, p-STAT3/6 were also activated at 30 min and 1 h after MB49-derived exosomes stimulation, however the underlying mechanism needs further investigation. To further confirm that the inhibition of PTEN by miR-1231-5p or miR-92b-3p in MB49-derived exosomes, we stimulated BMDMs with either miR-1231-5p mimic or miR-92b-3p mimic or each inhibitor with MB49-derived exosomes, respectively. We observed that the PTEN protein level was clearly decreased by miR-1231-5p mimic or miR-92b-3p mimic treatment, which was comparable with MB49-derived exosomes stimulation. Similarly, p-PI3K/p-AKT/p-STAT3/6 protein levels were increased by miR-1231-5p mimic or miR-92b-3p mimic treatment. In addition, MB49-derived exosomes mediated PTEN protein downregulation and p-PI3K/p-AKT/p-STAT3/6 upregulation were reversed by supplementation of either miR-1231-5p inhibitor or miR-92b-3p inhibitor (Fig. 6B). Collectively, these data suggested that miR-1231-5p and miR-92b-3p contained in MB49-derived exosomes play a key role in activating PTEN/AKT/STAT3/6 pathways.

In summary, we identified MB49-derived exosomal miRNAs could induce macrophage polarization to immunosuppressive phenotype by activating PTEN/AKT/STAT3/6 pathways, thus contributing to the establishment of immunosuppressive TME to facilitate tumor growth.

## Discussion

Dynamic and mutual crosstalk between heterogeneous tumor cells and stromal cells including immune cells plays a vital role in tumor formation and progression. In TME, tumor cells manipulate other stromal cells by a variety of signaling networks and molecules to facilitate their growth, invasion and metastasis [5]. Macrophages are heterogeneous and their phenotype and functions are regulated by the surrounding micro-environment [28]. As the major immune cells in TME, TAM have reported to have distinct functions during different stages of tumor progression, including tumor killing, angiogenesis, promoting tumor invasion, metastasis and mediating chemoresistance [29–31]. Therefore, it is of particular importance to investigate the mechanism of tumor cells regulating macrophage polarization. In the present study, we identified that MB49-derived exosomes contribute to immunosuppressive phenotype and function of TAM polarization by activating PTEN/AKT/STAT3 signaling pathways, thus promoting bladder cancer growth. Targeting MB49-derived exosomes release could inhibit macrophage M2 differentiation and increase T cells proliferation to impede tumor cell growth, therefore this study sheds new light on bladder cancer treatment.

It is well documented that tumor cells utilized soluble molecules like cytokines or metabolites to induce macrophage polarization in TME [14]. Recently, exosomes carrying genetic information including proteins, miRNAs have been suggested to play an essential role in affecting the functions of target cells by mediating the information transmission and exchange of material [18]. Therefore, tumor cells derived exosomes may contribute to the recruitment and reprogramming of TAM. A recent study reported that exosomes–transmitted long non-coding RNA PTENP1 suppresses bladder cancer progression by suppression PTEN expression [32]. Similarly, in present study, we found MB49-derived exosomes also suppress PTEN expression and induce BMDM M2 differentiation, thus contributing to immunosuppressive TME and facilitating tumor growth. Additionally, inhibition of exosomes generation or release by GW4869 significantly reduces the percentage of M2 macrophage as well as tumor growth in mice subcutaneous tumor model.

Exosomal miRNAs have been demonstrated to interfere with tumor immunity and the microenvironment to regulate cancer progression [26]. As described in our data, 246 miRNAs were identified in MB49-derived exosomes and associated with a variety of cancer related signaling pathways, like PI3K-AKT pathway. Two miRNAs, miR-92b-3p and miR-1231-5p, were found to inhibit *Pten* expression and AKT activation. It has been reported miR-92a-3p could promotes the proliferation, migration and invasion of esophageal squamous cell cancer (ESCC) by regulating PTEN [33], but its function on macrophage polarization is still not investigated. In addition, the effect of miR-1231-5p in PTEN inhibition and macrophage function needs further investigation. Since AKT activation is required for macrophage M2 polarization [34, 35], in current study we found MB49-derived exosomes could activate both AKT and STAT3/6 pathways to induce macrophage M2 polarization. However, the mechanism of activation of STAT3/6 pathway needs further exploration. Similarly, it has been demonstrated that bladder cancer cell line T24 derived exosomal miR-21 could also induce THP‑1 cell M2 differentiation by activation AKT-STAT3 pathway [22]. Here in this study, we confirmed that MB49 cells derived exosomal miRNAs: miR-1231-5p and miR-92b-3p could inhibit PTEN expression and activate PI3K/AKT-STAT3/6 pathway to induce BMDM M2 differentiation. However, the effect of other factors contained in MB49-derived exosomes on macrophage M2 polarization is still need to be further investigated. Moreover, miR-1231-5p and miR-92b-3p may be potential targets for bladder cancer therapy by suppressing macrophage M2 differentiation and reversing immunosuppressive TME.

An increasing body of evidence has demonstrated that TAM mediated immunosuppressive TME contributes to dampen adaptive anti-tumor immune responses [11]. In current study, we found MB49-derived exosomes could induce upregulation of immunosuppressive cytokines *Il10*, and *Tgfb* in BMDM. Moreover, TAM could also cause metabolic starvation of T cells by the activity of arginase and/or by production of immunosuppressive metabolites by indoleamine 2,3-dioxygenase 1/2 (IDO1/2) pathway [11, 36]. Indeed, we also observed that MB49-derived exosomes stimulated BMDM could hinder both CD4^+^ and CD8^+^ T cell proliferation, which can be partially rescued by GW4869 treatment. Furthermore, GW4869 treatment could also increase the percentage of CD4^+^ and CD8^+^ T cells and impede tumor growth in mice subcutaneous tumor model. These results indicate that MB49-derived exosomes reprogram macrophage into immunosuppressive phenotype and function, thus contributing to immunosuppressive TME and facilitating tumor progression.

## Conclusion

In summary, this study further confirmed bladder cancer cells derived exosomes contribute to the establishment of immunosuppressive TME and facilitate tumor progression. The exosomal miR-92b-3p and miR-1231-5p may play a vital role in activation of AKT/STAT3/6 signaling pathway and inducing macrophages immunosuppressive differentiation. In addition, hindrance of exosomes generation or release by GW4869 inhibited bladder tumor cell growth by reversing the immunosuppressive TME.

## Supplementary Information

**Additional file 1**. **Table S1:** The sequences of primers used in this study.

**Additional file 2**. **Table S2:** Antibodies used in this study.

**Additional file 3**. **Table S3:** Sequences of miRNA mimics

**Additional file 4**. **Table S4:** Sequences of miRNA inhibitors.

## Data Availability

All data generated or analyzed during this study are included in this article.

## Abbreviations

TAM: Tumor associated macrophages
TME: Tumor microenvironment
PTEN: Phosphate and tension homology deleted on chromosome ten
STAT3: Signal transducer and activator of transcription 3
STAT6: Signal transducer and activator of transcription 6
MIBC: Muscle invasive bladder cancer
CD206: Cluster of differentiation 206
CD163: Cluster of differentiation 163
TGF-β: Tumor growth factor beta
miR-21: MicroRNA 21
PI3K: Phosphatidylinositol 3-kinase
BMDM: Bone marrow-derived macrophages
IL-10: Interleukin-10
Inos: Inducible nitric oxide synthase

## References

[1] Tran L, Xiao JF, Agarwal N, Duex JE, Theodorescu D (2021). Advances in bladder cancer biology and therapy. Nat Rev Cancer.

[2] Babjuk M, Burger M, Compérat EM, Gontero P, Mostafid AH, Palou J, van Rhijn BWG, Rouprêt M, Shariat SF, Sylvester R (2019). European Association of Urology Guidelines on Non-muscle-invasive Bladder Cancer (TaT1 and Carcinoma In Situ) - 2019 Update. Eur Urol.

[3] Patel VG, Oh WK, Galsky MD (2020). Treatment of muscle-invasive and advanced bladder cancer in 2020. CA A Cancer J Clin.

[4] Witjes JA, Bruins HM, Cathomas R, Compérat EM, Cowan NC, Gakis G, Hernández V, Linares Espinós E, Lorch A, Neuzillet Y (2021). European association of urology guidelines on muscle-invasive and metastatic bladder cancer: summary of the 2020 guidelines. Eur Urol.

[5] Baghban R, Roshangar L, Jahanban-Esfahlan R, Seidi K, Ebrahimi-Kalan A, Jaymand M, Kolahian S, Javaheri T, Zare P (2020). Tumor microenvironment complexity and therapeutic implications at a glance. Cell Commun Signal.

[6] Binnewies M, Roberts EW, Kersten K, Chan V, Fearon DF, Merad M, Coussens LM, Gabrilovich DI, Ostrand-Rosenberg S, Hedrick CC (2018). Understanding the tumor immune microenvironment (TIME) for effective therapy. Nat Med.

[7] Mantovani A, Schioppa T, Porta C, Allavena P, Sica A (2006). Role of tumor-associated macrophages in tumor progression and invasion. Cancer Metastasis Rev.

[8] Chanmee T, Ontong P, Konno K, Itano N (2014). Tumor-associated macrophages as major players in the tumor microenvironment. Cancers.

[9] Yang L, Zhang Y (2017). Tumor-associated macrophages: from basic research to clinical application. J Hematol Oncol.

[10] Di Caro G, Cortese N, Castino GF, Grizzi F, Gavazzi F, Ridolfi C, Capretti G, Mineri R, Todoric J, Zerbi A (2016). Dual prognostic significance of tumour-associated macrophages in human pancreatic adenocarcinoma treated or untreated with chemotherapy. Gut.

[11] Mantovani A, Marchesi F, Malesci A, Laghi L, Allavena P (2017). Tumour-associated macrophages as treatment targets in oncology. Nat Rev Clin Oncol.

[12] Wang J, Li D, Cang H, Guo B (2019). Crosstalk between cancer and immune cells: Role of tumor-associated macrophages in the tumor microenvironment. Cancer Med.

[13] Zhou J, Tang Z, Gao S, Li C, Feng Y, Zhou X (2020). Tumor-Associated Macrophages: Recent Insights and Therapies. Front Oncol.

[14] Pan Y, Yu Y, Wang X, Zhang T (2020). Tumor-Associated Macrophages in Tumor Immunity. Front Immunol.

[15] Han C, Zhang C, Wang H, Zhao L (2021). Exosome-mediated communication between tumor cells and tumor-associated macrophages: implications for tumor microenvironment. Oncoimmunology.

[16] Mantovani A, Allavena P (2015). The interaction of anticancer therapies with tumor-associated macrophages. J Exp Med.

[17] Wortzel I, Dror S, Kenific CM, Lyden D (2019). Exosome-mediated metastasis: communication from a distance. Dev Cell.

[18] Kalluri R, LeBleu VS (2020). The biology, function, and biomedical applications of exosomes. Science (New York, NY).

[19] Mashouri L, Yousefi H, Aref AR, Ahadi AM, Molaei F, Alahari SK (2019). Exosomes: composition, biogenesis, and mechanisms in cancer metastasis and drug resistance. Mol Cancer.

[20] Zheng P, Luo Q, Wang W, Li J, Wang T, Wang P, Chen L, Zhang P, Chen H, Liu Y (2018). Tumor-associated macrophages-derived exosomes promote the migration of gastric cancer cells by transfer of functional Apolipoprotein E. Cell Death Dis.

[21] Binenbaum Y, Fridman E, Yaari Z, Milman N, Schroeder A, Ben David G, Shlomi T, Gil Z (2018). Transfer of miRNA in macrophage-derived exosomes induces drug resistance in pancreatic adenocarcinoma. Can Res.

[22] Lin F, Yin HB, Li XY, Zhu GM, He WY, Gou X (2020). Bladder cancer cell-secreted exosomal miR-21 activates the PI3K/AKT pathway in macrophages to promote cancer progression. Int J Oncol.

[23] Zhang Z, Jiang Z, Zhang Y, Zhang Y, Yan Y, Bhushan S, Meinhardt A, Qin Z, Wang M (2020). Corticosterone enhances the AMPK-mediated immunosuppressive phenotype of testicular macrophages during uropathogenic *Escherichia coli* induced orchitis. Front Immunol.

[24] Li W, Zhang X, Wu F, Zhou Y, Bao Z, Li H, Zheng P, Zhao S (2019). Gastric cancer-derived mesenchymal stromal cells trigger M2 macrophage polarization that promotes metastasis and EMT in gastric cancer. Cell Death Dis.

[25] Kwon Y, Kim M, Kim Y, Jung HS, Jeoung D (2020). Exosomal MicroRNAs as mediators of cellular interactions between cancer cells and macrophages. Front Immunol.

[26] Sun Z, Shi K, Yang S, Liu J, Zhou Q, Wang G, Song J, Li Z, Zhang Z, Yuan W (2018). Effect of exosomal miRNA on cancer biology and clinical applications. Mol Cancer.

[27] Lee YR, Chen M, Pandolfi PP (2018). The functions and regulation of the PTEN tumour suppressor: new modes and prospects. Nat Rev Mol Cell Biol.

[28] Shapouri-Moghaddam A, Mohammadian S, Vazini H, Taghadosi M, Esmaeili SA, Mardani F, Seifi B, Mohammadi A, Afshari JT, Sahebkar A (2018). Macrophage plasticity, polarization, and function in health and disease. J Cell Physiol.

[29] Vesely MD, Kershaw MH, Schreiber RD, Smyth MJ (2011). Natural innate and adaptive immunity to cancer. Annu Rev Immunol.

[30] Noy R, Pollard JW (2014). Tumor-associated macrophages: from mechanisms to therapy. Immunity.

[31] Lin Y, Xu J, Lan H (2019). Tumor-associated macrophages in tumor metastasis: biological roles and clinical therapeutic applications. J Hematol Oncol.

[32] Zheng R, Du M, Wang X, Xu W, Liang J, Wang W, Lv Q, Qin C, Chu H, Wang M (2018). Exosome-transmitted long non-coding RNA PTENP1 suppresses bladder cancer progression. Mol Cancer.

[33] Li X, Guo S, Min L, Guo Q, Zhang S (2019). miR-92a-3p promotes the proliferation, migration and invasion of esophageal squamous cell cancer by regulating PTEN. Int J Mol Med.

[34] Vergadi E, Ieronymaki E, Lyroni K, Vaporidi K, Tsatsanis C (2017). Akt Signaling pathway in macrophage activation and M1/M2 polarization. J Immunol (Baltimore, Md: 1950).

[35] Wang D, Wang X, Si M, Yang J, Sun S, Wu H, Cui S, Qu X, Yu X (2020). Exosome-encapsulated miRNAs contribute to CXCL12/CXCR4-induced liver metastasis of colorectal cancer by enhancing M2 polarization of macrophages. Cancer Lett.

[36] Murray PJ, Allen JE, Biswas SK, Fisher EA, Gilroy DW, Goerdt S, Gordon S, Hamilton JA, Ivashkiv LB, Lawrence T (2014). Macrophage activation and polarization: nomenclature and experimental guidelines. Immunity.

