# Supplementary material for: Cancer derived exosomes induce macrophages immunosuppressive polarization to promote bladder cancer progression
Source: Cell Commun Signal. 2021 Sep 14;19:93. doi: 10.1186/s12964-021-00768-1 (PMC8439012; doi:10.1186/s12964-021-00768-1)
Supplement: Supplementary file 2 — Additional file 1. Table S1: The sequences of primers used in this study. [file 12964_2021_768_MOESM2_ESM.docx]

**Supplementary table 1. Primer list.**

| Gene | Primer sequence |
| --- | --- |
| *Il10* | Forward: 5'-GACTTTAAGGGTTACCTGGGTTG-3'  Reverse: 5'-TCACA TGCGCCTTGA TGTCTG-3' |
| *Tgfβ* | Forward: 5'-GGTACCTGAACCCGTGTTGCT-3'  Reverse:5'-TGTTGCTGTATTTCTGGTAACAGCTC-3' |
| *Cd206* | Forward: 5'- GGGACTCTGGATTGGACTCA-3'  Reverse: 5'- CCAGGCTCTGATGATGGACT-3' |
| *Inos* | Forward: 5'-CTGCAGCACTTGGATCAGGAACCTG-3'  Reverse: 5'-GGAGTAGCCTGTGTGCACCTGGAA -3' |
| *Actin* | Forward: 5'- AACAGTCCGCCTAGAAGCAC -3'  Reverse: 5'- CGTTGACATCCGTAAAGACC -3' |
