# Supplementary material for: Cancer derived exosomes induce macrophages immunosuppressive polarization to promote bladder cancer progression
Source: Cell Commun Signal. 2021 Sep 14;19:93. doi: 10.1186/s12964-021-00768-1 (PMC8439012; doi:10.1186/s12964-021-00768-1)
Supplement: Supplementary file 3 — Additional file 2. Table S2: Antibodies used in this study. [file 12964_2021_768_MOESM3_ESM.docx]

**Supplementary table 2. Antibody list.**

| **Antibodies** | **Company** | **Lot No.** | [**concentration**](javascript:;) |
| --- | --- | --- | --- |
| HSP90 | Cell Signaling Technology | 4877S | 1:1000 |
| p-AKT | Cell Signaling Technology | 13038S | 1:1000 |
| p-STAT3 | Cell Signaling Technology | 9145S | 1:1000 |
| p-STAT6 | Cell Signaling Technology | 9361S | 1:1000 |
| p-PI3K | Cell Signaling Technology | 17366S | 1:1000 |
| PTEN | Abcam | ab32199 | 1:1000 |
| CD63 | Santa Cruz Biotechnology | sc-15363 | 1:1000 |
| Actin | Proteintech | BC00249 | 1:2000 |
| anti-Rabbit IgG | Thermo Fisher | A-31573 | 1:5000 |
| F4/80-APC/Cy7 | Biolegend | 123118 | 1:100 |
| CD206-percp5.5 | Biolegend | 141716 | 1:100 |
| CD4-APC/Cy7 | Biolegend | 100414 | 1:100 |
| CD8-percp5.5 | Biolegend | 100732 | 1:100 |
| CD11b-FITC | Becton, Dickinson | 553310 | 1:100 |
