# Supplementary material for: Cancer derived exosomes induce macrophages immunosuppressive polarization to promote bladder cancer progression
Source: Cell Commun Signal. 2021 Sep 14;19:93. doi: 10.1186/s12964-021-00768-1 (PMC8439012; doi:10.1186/s12964-021-00768-1)
Supplement: Supplementary file 4 — Additional file 3. Table S3: Sequences of miRNA mimics [file 12964_2021_768_MOESM4_ESM.docx]

**Supplementary table 3. Sequences of miRNA mimics.**

| Gene name | Sense (5'-3') | Antisense (5'-3') |
| --- | --- | --- |
| mmu-miR-1231-5p | UCUGGGCAGAGCUGCAGGAGAGA | UCUCCUGCAGCUCUGCCCAGAUU |
| mmu-miR-92b-3p | UAUUGCACUCGUCCCGGCCUCC | AGGCCGGGACGAGUGCAAUAUU |
| mimic NC | UUCUCCGAACGUGUCACGUTT | ACGUGACACGUUCGGAGAATT |
