# Supplementary material for: Cancer derived exosomes induce macrophages immunosuppressive polarization to promote bladder cancer progression
Source: Cell Commun Signal. 2021 Sep 14;19:93. doi: 10.1186/s12964-021-00768-1 (PMC8439012; doi:10.1186/s12964-021-00768-1)
Supplement: Supplementary file 5 — Additional file 4. Table S4: Sequences of miRNA inhibitors. [file 12964_2021_768_MOESM5_ESM.docx]

**Supplementary table 4. Sequences of miRNA inhibitors.**

| Gene name | sense（5'-3'） |
| --- | --- |
| mmu-miR-1231-5p | UCUCUCCUGCAGCUCUGCCCAGA |
| mmu-miR-92b-3p | GGAGGCCGGGACGAGUGCAAUA |
| inhibitor NC | CAGUACUUUUGUGUAGUACAA |
